# CASE REPORT Rotational Vascularized Tibiaplasty After Oncologic Resection and Major Wound Healing Problems: A Novel Technique

**Published:** 2013-08-19

**Authors:** Cornelius Dieter Schubert, Frank J. Frassica, Samer Attar, E. Gene Deune

**Affiliations:** Department of Orthopaedic Surgery, The Johns Hopkins University, Baltimore, Md

## Abstract

**Objective:** To describe a novel method to reconstruct, with a vascularized rotational tibiaplasty, a complex femoral defect in an adolescent. **Methods:** After a femoral osteosarcoma resection, allograft reconstruction, and chemotherapy, an 11-year-old girl developed recurrent thigh wound infections and femoral allograft osteomyelitis despite multiple operative interventions. At the age of 13, she presented to our center with a complex right thigh wound and an unstable lower extremity secondary to a segmental femoral loss. To reestablish thigh stability and function and to avoid amputation at the hip, the authors performed a rotational vascularized tibiaplasty. The tibia was rotated 180° with the pivot at the knee. The distal tibia was internally stabilized to the residual proximal femur. **Results:** Ten years later, the patient had a stable thigh, a functional hip, no evidence of infection or sarcoma, and a Toronto Extremity Salvage Score of 92.5 (minimal disability). **Conclusions:** In this patient, the tibial rotationplasty provided a vascularized bone strut mimicking the resected femur; saved the hip; obviated an allograft bone; and created a functional, biologic, stable, and durable thigh that allowed full weight bearing on a prosthesis, with a low physical disability level. We conclude that, for patients with complex femoral defects, a vascularized rotational tibiaplasty should be considered a feasible option before amputation.

A large segmental bone loss with or without soft tissue loss can be reconstructed with a rotational vascularized bone (tibial or fibular) autograft from the ipsilateral lower leg, known as a rotationplasty. Since Borggreve^1^ first described this functional limb-sparing procedure in 1930, multiple modifications have evolved^2^^-^4 for treating posttraumatic osteomyelitis,^5^ for treating endoprosthesis infection or failure,^6^^,^^7^ for oncologic tumor resections,^6^ or for extremity salvage to avoid amputation.

## PATIENT HISTORY

**Figure 1:**
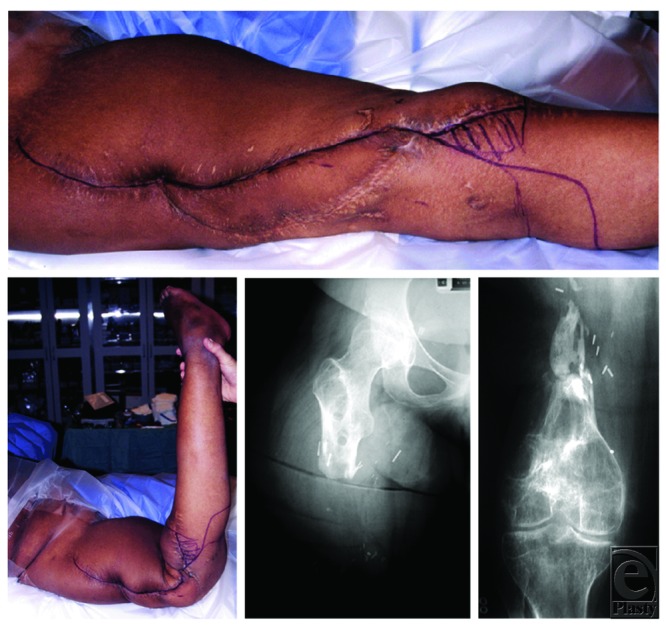
Clinical preoperative images. (*Top*) Extensive scarring. (*Bottom left*) An unstable thigh with 90° of dorsal extension in the distal thigh secondary to the lack of an intact femur. The thigh is freely mobile to deforming forces. Anteroposterior radiographs of the right hip (*bottom, middle*) and the right knee joint (*bottom, right*) display the major bony defect.

An 11-year-old girl with a right femoral osteosarcoma underwent resection, adjuvant chemotherapy, and immediate reconstruction with an allograft femur and a rectus abdominis free flap at another institution. Her postoperative course was complicated by persistent wound infections and allograft osteomyelitis. She presented to the authors’ institution at the age of 13 with a complex right thigh wound and an unstable lower extremity (Fig 1). The allograft femur was removed and replaced with a tobramycin-impregnated cement spacer. She subsequently underwent intramedullary rodding with methyl methacrylate, placement of an Ilizarov external fixator, and placement of a cortical strut allograft bone to the proximal femur. She again developed purulent drainage, requiring additional debridements and fixator removal. All additional attempts of lengthening were abandoned. To avoid allograft tissue placement, further infections, or amputation at the hip and to re-establish a stable, functional thigh, the authors performed a rotational vascularized autologous tibiaplasty. We discussed the operative plans in great detail with the patient and her parent, and they decided to proceed with the reconstructive attempt.

## SURGICAL TECHNIQUE

**Figure 2:**
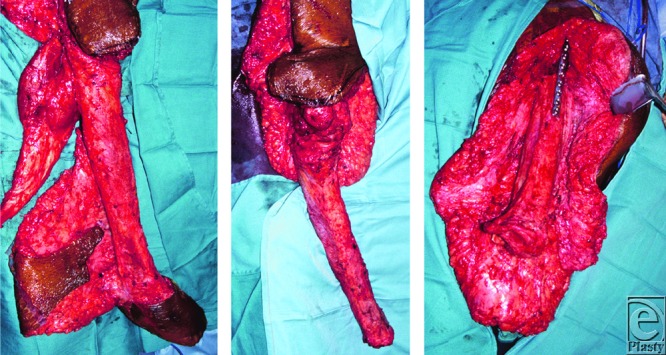
Intraoperative photographs. (*Left*) Before rotation, the vascularized tibiaplasty is shown with attached perfusion arteries and associated soft tissue. After amputation of the foot, excision of the fibula, and resection of the soleus and gastrocnemius muscles (*middle*), the tibia was rotated and internally fixed to the proximal femur (*right*).

A lateral thigh incision for the recipient tibia was made through previous incisions, followed by an anterior midline longitudinal incision from the knee to the ankle. Medial and lateral fasciocutaneous flaps were elevated (Fig 2, *left*). The soleus and the gastrocnemius muscles were resected, and the fibula was excised. The anterior and lateral compartment muscles were carefully dissected off the tibia, leaving only the anterior tibial, posterior tibial, and peroneal arteries, and the surrounding soft tissues attached to the tibia. Distally, the dorsalis pedis, peroneal, and posterior tibial arteries and veins were dissected and ligated at the ankle. The foot was amputated at the tibiotalar joint, and the tibia was converted into a vascularized bone flap (Fig 2, *middle*). The popliteal artery and veins were dissected free at the distal thigh and the popliteal fossa.

**Figure 3:**
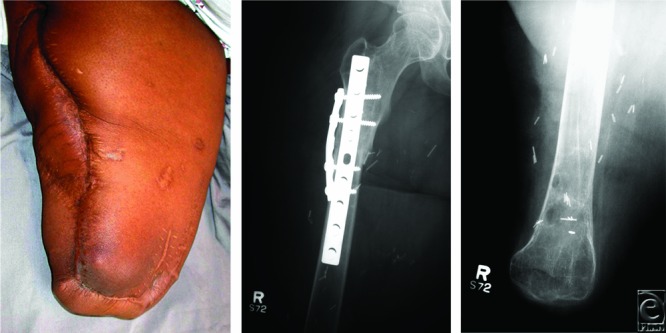
Thirty months after surgery. There has been excellent healing of the right thigh with a functional hip joint (*left*). Anteroposterior radiographs show osteosynthesis of the distal tibia and proximal femur (*middle*) and the proximal tibia serving as the distal tip of the rotationplasty (*right*).

The remnant distal femur was removed and disarticulated from the knee joint, which allowed the tibia to be rotated vertically. A soft tissue trough was created in the thigh, where the mid femur had been previously located. For proper length match, 10 cm of the distal tibia were removed. Osteosynthesis between the proximal femur and the distal tibia was achieved with an anterior internal 9-hole, 4.5-mm, narrow dynamic compression plate and a 5-hole, 4.5-mm, narrow dynamic compression plate (Synthes, West Chester, PA) placed 90° and lateral to the first plate (Fig 3, *middle*). The soft tissue defect was reconstructed with local muscle and tissue. Wounds were closed in multiple layers over 2 drains. The patient was discharged on postoperative day 11. She returned 2 months after surgery for an operative debridement of a small infected distal thigh seroma that grew *Enterococcus faecalis*. She was placed on intravenous and oral antibiotics.

## ASSESSMENT OF FUNCTIONAL OUTCOME

The patient's functional outcome was assessed with the Toronto Extremity Salvage Score (TESS). The TESS was developed specifically for patients with extremity sarcomas.^8^ It is a questionnaire designed to measure physical function and disability from the patient's perspective at a single point in time. The TESS questionnaire for disabilities of the lower extremity includes 30 questions, each rated on a 5-point Likert scale. The questions relate to the patient's level of difficulty performing routine daily activities, such as dressing, grooming, mobility, work, sports, and leisure. The total TESS score is then calculated as a percentage, ranging from 0 to 100.^9^ Higher scores indicate less difficulty with activities of daily living.

## RESULTS

At 3.5 years after surgery, she had an unstable wound at the distal lateral tibial condyle that prevented her wearing her prosthesis. She underwent resection of this wound and soft tissue coverage by a rhomboid fasciocutaneous flap (6 × 6 cm). The wounds healed without further complications (Fig 3).

At 10 years after her rotationplasty, the patient had no sarcoma recurrence and no further infections. Her TESS was 92.5, indicating a low level of physical disability. She was able to bear full weight on her prosthesis, was leading an active and psychosocially normal life, and was happy she had had no subsequent infections.

## DISCUSSION

Limb-sparing surgery for extremity sarcomas requires a coordinated preoperative, intraoperative, and postoperative approach between plastic and orthopedic surgery. When major wide-margin bone resection is required in the lower extremity, operative options include the Borggreve-Van Nes rotationplasty with functional reconstruction of the knee joint,^1^^,^^4^^,^^10^ an alloplastic prosthesis with soft tissue reconstruction using pedicled muscle flaps^11^ or free flaps, and a rotational vascularized tibiaplasty. A Borggreve-van Nes rotationplasty would have been technically more demanding and would have had substantially higher risks for failure because the severe scarring from her previous extensive surgeries would have prevented proper proximal advancement of the tibia for a pedicled vascularized flap rotationplasty. Her previous rectus abdominis free flap was anastomosed to her femoral artery. A second free flap consequently presented unacceptably high technical challenges and complication risks and could have negatively affected the option of using the tibia as a salvage procedure.

The rotational tibiaplasty is preferably suited for patients with large, complicated defects affecting the entire femur and knee joint. The tibia is well suited to replace the femur in this clinical setting. In our patient, the tibia was free of tumor and infection and provided healthy and well-vascularized autologous bony tissue sufficient enough to support her weight with her prosthesis once healing occurred.

Other alternatives to rotationplasty are associated with disadvantages and/or fewer benefits. Amputation at the hip is associated with higher rates of postoperative complications, phantom pain, substantial patient dissatisfaction, and difficulties when using a prosthesis.^12^ According to Daigeler et al,^12^ amputation should be considered the last treatment option for severe infections or extremity malignancies. Compared with rotationplasty, endoprosthetic replacements have resulted in more restricted daily activities because of pain,^13^ less participation in hobbies,^13^ and lower TESS.^14^ An endoprosthetic replacement of the femur would have been inappropriate because of the patient's history of multiple infections and wound-healing problems. In this young patient, the authors considered the rotationplasty the best option for medical and psychologic reasons. Rodl et al^15^ analyzed the life contentment, occupational situation, marriage status, and quality of life of patients who had rotationplasty and showed no reduction in psychosocial adaption compared with healthy people.

## CONCLUSION

In this patient, the tibial rotationplasty provided a vascularized structural bone strut mimicking the resected femur; saved the hip; obviated an allograft bone; and created a functional, biologic, stable, and durable thigh that allowed full weight bearing on a prosthesis, with a low physical disability level.

## References

[1] Borggreve J (1930). Kniegelenksersatz durch das in der Beinlängsachse um 180 gedrehte Fussgelenk. Arch Orthop Unfall-Chir.

[2] Kristen H, Knahr K, Salzer M (1975). Atypical amputations of bone tumors of the lower extremity (author's transl) [in German]. Arch Orthop Unfallchir.

[3] Merkel KD, Reinus WR, Miller G, Koudsi B (1997). Modification of the Van Nes rotationplasty: report of a case. Clin Orthop Relat Res.

[4] Van Nes CP (1950). Rotation-plasty for congenital defects of the femur: making use of the ankle of the shortened limb to control the knee joint of the prosthesis. J Bone Joint Surg Br.

[5] Krettek C, Lewis DA, Miclau T, Schandelmaier P, Lobenhoffer P, Tscherne H (1997). Rotationplasty for the treatment of severe bone loss and infection of the distal end of the femur. A case report. J Bone Joint Surg Am.

[6] Fuchs B, Sim FH (2004). Rotationplasty about the knee: surgical technique and anatomical considerations. Clin Anat.

[7] Wicart P, Mascard E, Missenard G, Dubousset J (2002). Rotationplasty after failure of a knee prosthesis for a malignant tumour of the distal femur. J Bone Joint Surg Br.

[8] Davis AM, Wright JG, Williams JI, Bombardier C, Griffin A, Bell RS (1996). Development of a measure of physical function for patients with bone and soft tissue sarcoma. Qual Life Res.

[9] Davis AM, Sennik S, Griffin AM (2000). Predictors of functional outcomes following limb salvage surgery for lower-extremity soft tissue sarcoma. J Surg Oncol.

[10] Dumont CE, Schuster AJ, Freslier-Bossa M (2010). Borggreve-Van Nes rotationplasty for infected knee arthroplasty: a case report. Acta Orthop.

[11] Steinau HU, Daigeler A, Langer S (2010). Limb salvage in malignant tumors. Semin Plast Surg.

[12] Daigeler D, Lehnhardt M, Khadra A (2009). Proximal major limb amputations—a retrospective analysis of 45 oncological cases. World J Surg Oncol.

[13] Hillmann A, Hoffmann C, Gosheger G, Krakau H, Winkelmann W (1999). Malignant tumor of the distal part of the femur or the proximal part of the tibia: endoprosthetic replacement or rotationplasty: functional outcome and quality-of-life measurements. J Bone Joint Surg Am.

[14] Jones KB, Griffin AM, Chandrasekar CR (2011). Patient-oriented functional results of total femoral endoprosthetic reconstruction following oncologic resection. J Surg Oncol.

[15] Rodl RW, Pohlmann U, Gosheger G, Lindner NJ, Winkelmann W (2002). Rotationplasty—quality of life after 10 years in 22 patients. Acta Orthop Scand.

